# The Effect of SERCA Activation on Functional Characteristics and Signaling of Rat Soleus Muscle upon 7 Days of Unloading

**DOI:** 10.3390/biom13091354

**Published:** 2023-09-06

**Authors:** Kristina A. Sharlo, Irina D. Lvova, Sergey A. Tyganov, Ksenia A. Zaripova, Svetlana P. Belova, Tatiana Y. Kostrominova, Boris S. Shenkman, Tatiana L. Nemirovskaya

**Affiliations:** 1 Myology Laboratory, Institute of Biomedical Problems, RAS (Russian Academy of Sciences), Moscow 123007, Russia; sharlokris@gmail.com (K.A.S.); irrrra1@yandex.ru (I.D.L.); sentackle@yandex.ru (S.A.T.); katsu.no.himitsu@gmail.com (K.A.Z.); swetbell@mail.ru (S.P.B.); bshenkman@mail.ru (B.S.S.); 2 Department of Anatomy, Cell Biology and Physiology, Indiana University School of Medicine-Northwest, Gary, IN 46202, USA; tkostrom@iun.edu

**Keywords:** soleus muscle unloading, muscle atrophy, ATP, AMPK, MuRF1, MAFbx

## Abstract

Skeletal muscle abnormalities and atrophy during unloading are accompanied by the accumulation of excess calcium in the sarcoplasm. We hypothesized that calcium accumulation may occur, among other mechanisms, due to the inhibition of sarco/endoplasmic reticulum Ca^2+^-ATPase (SERCA) activity. Consequently, the use of the SERCA activator will reduce the level of calcium in the sarcoplasm and prevent the negative consequences of muscle unloading. Wistar rats were randomly assigned into one of three groups (eight rats per group): control rats with placebo (C), 7 days of unloading/hindlimb suspension with placebo (7HS), and 7 days of unloading treated with SERCA activator CDN1163 (7HSC). After seven days of unloading the soleus muscle, the 7HS group displayed increased fatigue in the ex vivo test, a significant increase in the level of calcium-dependent CaMK II phosphorylation and the level of tropomyosin oxidation, as well as a decrease in the content of mitochondrial DNA and protein, slow-type myosin mRNA, and the percentage of slow-type muscle fibers. All of these changes were prevented in the 7HSC group. Moreover, treatment with CDN1163 blocked a decrease in the phosphorylation of p70S6k, an increase in eEF2 phosphorylation, and an increase in MuRF-1 mRNA expression. Nevertheless, there were no differences in the degree of fast and slow muscle fiber atrophy between the 7HS and 7HSC groups. Conclusion: SERCA activation during 7 days of unloading prevented an increase in soleus fatigue, the decrease of slow-type myosin, mitochondrial markers, and markers of calcium homeostasis but had no effect on muscle atrophy.

## 1. Introduction

A number of skeletal muscle abnormalities, including prolonged hypokinesia, gravitational unloading, and limb immobilization, as well as prolonged lack of physical activity, can lead to skeletal muscle atrophy due to an imbalance between protein synthesis and protein degradation. During unloading, the content of mitochondrial proteins and DNA in muscles decreases concurrently with the development of atrophy [1,2,3]. Changes in the expression pattern of myosin genes lead to an increase in the proportion of “fast” glycolytic fibers with weak resistance to fatigue [4,5,6]. As a result, there is a decrease in muscle performance.

Several models of muscle unloading display increased accumulation of calcium in the sarcoplasm [7,8,9]. This effect is associated with Na, K-ATPase dysfunction, and depolarization of sarcolemma at the early stages of unloading, which results in the activation of dihydropyridine channels and the entry of calcium into the sarcoplasm through ryanodine receptors [10]. Excessive accumulation of calcium in the sarcoplasm is observed in a number of other pathological conditions, for example, in Duchenne muscular dystrophy and during muscle aging [11,12]. A high concentration of calcium in the sarcoplasm may stimulate the activation of calcium-dependent calpain proteases and increased proteolysis [13,14], as well as disruption of mitochondrial function [15]. Abnormalities in calcium homeostasis in skeletal muscle result in changes in gene expression, including markers of proteolysis as well as genes regulating the muscle fiber type (“fast” or “slow”) [16,17].

Sarco/endoplasmic reticulum Ca^2+^-ATPase (SERCA) plays a key role in the removal of calcium from the sarcoplasm by pumping calcium ions into the sarcoplasmic reticulum (SR). During skeletal muscle unloading, the function of SERCA is impaired [18]. Likewise, a decrease in the pumping of calcium ions into the SR is observed in skeletal muscle during immobilization and denervation [19,20]. It was previously reported that skeletal muscle unloading results in changes in the expression pattern of SERCA isoforms, which can lead to changes in the redistribution of calcium ions between the SR and sarcoplasm [21,22,23].

Excessive accumulation of calcium in the sarcoplasm can also lead to the accumulation of reactive oxygen species (ROS) and of the disruption of ryanodine channel functioning. This results in the depletion of the SR calcium depot and increased muscle fatigability. Similar changes were described in skeletal muscle during aging, as well as in the muscle of some transgenic animals [24,25,26,27,28]. Since excessive accumulation of calcium and ROS in the sarcoplasm is also observed during skeletal muscle unloading [7,29], it cannot be ruled out that calcium-dependent processes contribute to the increased muscle fatigability under these conditions. This suggestion is supported by a recent study that reported a functional interaction between SERCA and mitochondrial oxidative phosphorylation [30]. It shows the prevention of the development of soleus muscle fatigue when the SERCA activator is administered to mice.

We hypothesized that during soleus muscle unloading, there is a decrease in SERCA functions leading to the excessive accumulation of calcium ions in the sarcoplasm. Consecutively, increased calcium activates signaling pathways, triggering proteolysis processes and changes in the myosin phenotype from “slow” to “fast”, resulting in reduced fatigue resistance. In humans, the soleus muscle is involved in walking and running, and it is also responsible for maintaining the vertical position of the body. It is also very sensitive to unloading and shows fast loss of mass and functions [31]. This is why the soleus muscle was chosen for the experiments in the current study. A specific SERCA activator CDN1163 was used to test a hypothesis that a decreased SERCA activity in skeletal muscle during unloading regulates cellular signaling pathways and a decrease in muscle contractile properties. If this hypothesis is correct, then activation of SERCA during unloading should lead to decreased muscle fatigue and should prevent unloading-induced changes in the myosin composition.

## 2. Materials and Methods

### 2.1. Animal Procedures

The experiments were performed at the Institute of Biomedical Problems, RAS, Russia. The Committee on Bioethics of the Russian Academy of Sciences reviewed and approved all animal experiments for this study (protocol 584; 31 May 2021). The internationally accepted regulations in compliance with ARRIVE guidelines [32] and rules of biomedical ethics were followed during this study in compliance with the principles and regulations described by Grundy [33].

Twenty-four male Wistar rats (two and a half months old) were randomly assigned to one of the three groups (eight animals per group): control rats with placebo (intraperitoneal injection of 400 mL 10% DMSO, 10% Tween 80 in physiological saline) (“C”), 7 days of unloading/hindlimb suspension with placebo (“7HS”), and 7 days of unloading with SERCA activator CDN1163 (intraperitoneal injection of 50 mg/kg of body weight per day in 400 mL 10% DMSO, 10% Tween 80 in physiological saline; “7HSC”) as previously described [34].

Hindlimb suspension was performed using a traction method of noninvasive tail-casting [35]. During unloading, rats were free to move around the cage using forelimbs and had food and water ad libitum. Animals were kept at 22 °C in a light-controlled environment (12:12 h light–dark cycle). At the completion of seven-day experiments, the rats were euthanized by the isoflurane inhalation anesthesia with subsequent cervical dislocation. The right soleus muscle was dissected, weighed, frozen in liquid nitrogen, and stored at −85 °C for the subsequent analyses.

### 2.2. Soleus Muscle Ex Vivo Fatigue Analysis

The detailed description of how soleus muscle ex vivo function was determined is described in our previous study [36]. Briefly, the soleus muscles were incubated for 15 min in a cooled Ringer–Krebs solution with constant perfusion with 95% carbogen (O2 + C2) and then they were placed into a test bath with a fixed temperature of 37 °C. The muscle was attached via tendons to a force sensor at one side and to the fixed hook at the other side. Optimal muscle length (L0) was determined using a digital caliper. After single contractions, tetanic isometric contractions were performed. The muscle was set to length L0, and it was electrically stimulated with a frequency of 40 Hz for 2 s. The maximum strength of the tetanic contraction was recorded. To test the fatigue index of the soleus muscle, a series of 20 tetanic contractions were performed during one minute with a pause of 1 s between contractions. For the fatigue index, the ratio of the force of contraction after 20 repetitions was normalized to the maximum contraction force measured during the entire test. Scores were normalized to the muscle physiological cross-sectional area (pCSA). 

### 2.3. Protein Extraction, Gel Electrophoresis, and Western Blot

Protein extraction, gel electrophoresis, and Western blot analysis were performed as previously described [37]. As a brief summary, total protein samples in Laemmli buffer were separated by 10% SDS-PAGE and transferred to nitrocellulose membranes (Bio-Rad Laboratories, Hercules, CA, USA) in Western Blot Transfer Buffer (25 mM Tris, 192 mM glycine, pH 8.3, 20% ethanol, 0.04% SDS) at 100 V and 4 °C in the mini-Trans-Blot system (Bio-Rad Laboratories, Hercules, CA, USA) for 120 min. The membranes were incubated with the following primary antibodies: total CaMKII (1:1000, CSB-PA061493) and p-(Thr180/Tyr-182) CaMKII (1:1000, CSB-PA283993) from Cusabio, Wuhan, China; p70S6k (1:1000, #9202), p-(T56)-eEF2 (1:1000, #2331), eEF2 (1:1000 #2332), p-(Ser 9)-GSK3b (1:1000, #9336), GSK3b (1:1000), MAP kinase p38 (1:500 #9212) from Cell signaling Technology, Inc, Danvers, MA, USA); p-(Thr 183, Tyr 185)-JNK1/2 (1:1000, 44-682G, Thermo Fisher Scientific, Waltham, MA, USA); tJNK (1:1000, SAB4200176, Sigma-Aldrich, Burlington, MA, USA); GAPDH (1:10,000, G 041, ABM, RVA, Canada); p-(Thr-389)-p70S6K (1:1000, sc-11759); tropomyosin (1:1000, sc-58868) from Santa Cruz Biotechnology, Dallas, TX, USA; p-(Thr180/Tyr182)-MAP kinase p38 (1:500, # GTX59567) from GeneTex, Irvine, CA, USA; and TOM20 4F3 from Bio-Rad, Germany. Secondary antibodies were horseradish peroxidase-conjugated goat anti-rabbit (1:30,000, #111-035-003, Jackson Immuno Research, West Grove, PA, USA) or goat anti-mouse (1:20,000, #1706516, Bio-Rad, Hercules, CA, USA). To determine the level of phosphorylation, phosphorylated protein content was normalized to the total protein content of the same protein.

### 2.4. Reverse Transcription and Real-Time PCR

To study gene expression and mitochondrial DNA content by real-time quantitative PCR, RNA and DNA were isolated from muscle tissue samples. For RNA isolation from skeletal muscles the HiPure Fibrous DNA/RNA Kit (Magen Biotechnology, Guangzhou, China) was used according to the manufacturer’s recommendations. RNA and DNA concentration analysis, cDNA synthesis and cDNA and DNA RT-PCRs were performed as previously described [36]. The PCR primers used in this study are listed in Table 1. PCR data were normalized for the expression of the “housekeeping” RPL19 gene (or RPL19 DNA content for DNA PCR). The data were calculated according to the method of Pfaffl et al. [38]. For each target gene, the PCR reaction was carried out at least 3 times.

### 2.5. Immunohistochemical Analyses

Transverse sections of frozen muscle 9 μm thick were made using cryotome (Leica Biosystems, Wetzlar, Germany). The sections were air-dried and stored at −20 °C. Before immunostaining, sections were thawed and rehydrated at room temperature in phosphate buffered saline (PBS) for 20 min. The protocol of immunostaining for the evaluation of the percent of the slow-type and fast-type myofibers and for the quantification of their cross-sectional areas (CSAs) was previously described [39]. The primary antibodies that were used were: anti-MyHC I(β) slow (1:100, Sigma, St. Louis, MO) and anti-MyHCs fast (1:60, DSMZ). Secondary antibodies were Alexa Fluor 488 and Alexa Fluor 546 (1:1000; Molecular Probes, Waltham, MA, USA).

The percent of NFATc1-positive myonuclei relative to the count of total myonuclei was determined using triple staining of the cryosections with anti-NFATc1 and anti-dystrophin antibodies, and with DAPI. DAPI-stained nuclei lying within sarcoplasm below the dystrophin layer were assumed to be myonuclei. After rehydration, muscle cryosections were placed for 1 h into a blocking solution (1% BSA—bovine serum albumin and 0.1% Tween 20 in PBS—phosphate-buffered saline, pH 7.4) at room temperature. Primary antibodies against NFATc1 (sc-1149, Santa Cruz Biotechnology, Dallas, TX, USA, 1:100) and against dystrophin (ab15277, Abcam, Waltham, MA, USA 1:100) in blocking solution were added 150 µL per slide with overnight incubation at 4 °C. A negative control (blocking solution without primary antibodies) was used to check for non-specific binding of secondary antibodies to the sections. The next day, the sections were washed with 200 µL of PBS per a glass slide 3 times for 5 min and 150 µL of secondary antibodies (1:500 goat anti-mouse, Alexa 488 and goat anti-rabbit, Alexa 546; Molecular Probes, Eugene, OR, USA) diluted in PBS were added at room temperature for 1 h. After incubation, sections were washed for 5 min with 200 µL of PBS and incubated for 20 min with DAPI (Molecular Probes, Eugene, OR, USA, 1:10,000 in PBS). The sections were washed from DAPI with 200 μL of PBS per glass slide 2 times for 5 min. Then, the sections were dried with filter paper, embedded in Vectashield fluorescence solution, and covered with a cover-slip with varnish fixation. Sections were analyzed using a Leica DMR Upright Microscope equipped with a Leica DC 300F camera (Leica Biosystems, Wetzlar, Germany). The photographs were analyzed using the ImageJ program.

### 2.6. Statistical Analysis

The data were checked for normal distribution by Shapiro–Wilk test. All data were normally distributed, therefore, an ANOVA was used with post hoc Tukey analysis for multiple comparisons. Differences were considered significant when *p* < 0.05. The data are presented as mean ± standard error of the mean relative to the control group. For QRT-PCR the data are presented as relative expression with mean value of the control group assigned to be 1.

## 3. Results

### 3.1. The Effects of Treatment with CDN1163 on Muscle Mass and Mechanical Characteristics during Unloading

**Figure 1:**
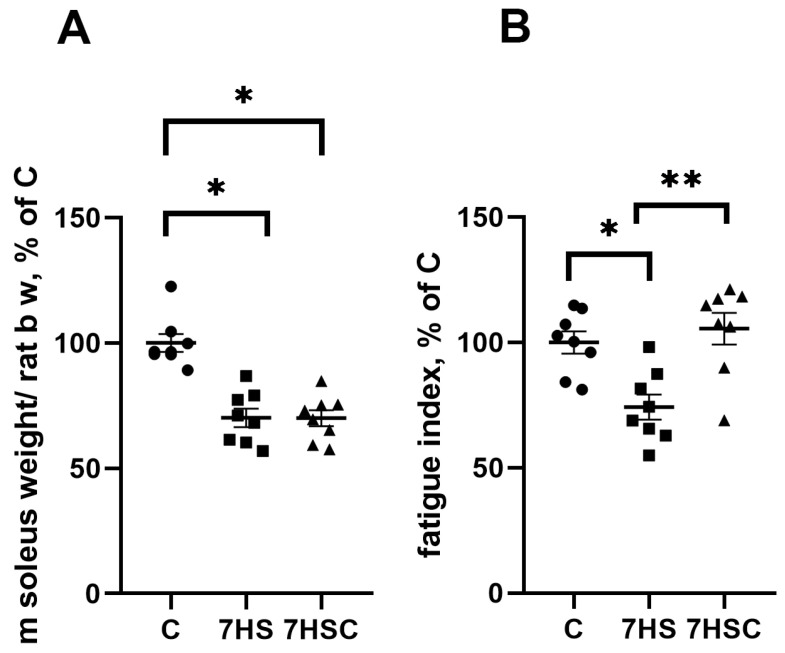
Soleus muscle weight normalized to rat total body weight (**A**) and soleus muscle fatigue index (**B**) in control rats (C), rats with 7 days of unloading (7HS), and 7 days of HS with CDN1163 (7HSC). N = 8. * indicates a significant difference from the control, ** indicates a significant difference from the HS group, *p* < 0.05.

After seven days of unloading, the soleus muscle mass was significantly decreased in both HS and 7HSC groups (67.5 ± 2.9 mg and 65.3 ± 4.2 mg, respectively) when compared with the control rats (109.4 ± 4.3 mg) (Figure 1A). At the same time, treatment with the CDN1163 prevented the unloading-induced decline of the muscle fatigue index (Figure 1). The HS group had a 17% decline in the fatigue index when compared with the control, and this decline was completely prevented in the 7HSC group (Figure 1).

### 3.2. The Effects of Treatment with CDN1163 on Calcium Signaling and Tropomyosin Oxidation during Unloading

**Figure 2:**
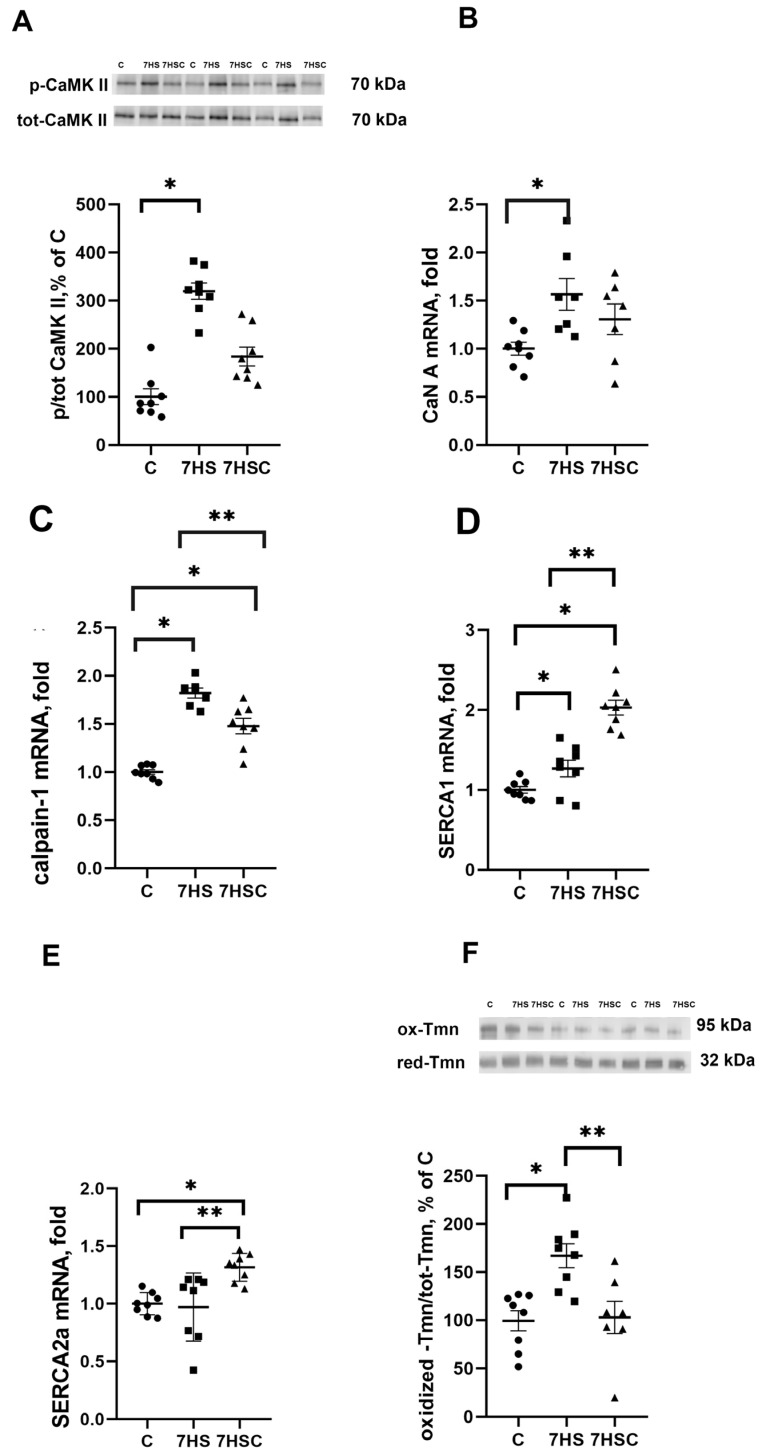
Evaluation of CaMKII phosphorylation (**A**), CaN A (**B**), calpain-1 (**C**), SERCA1 (**D**), and SERCA2 (**E**). mRNA expression, and oxidized tropomyosin content (**F**) in soleus muscle of control rats (C), rats with 7 days of unloading (7HS), and 7 days of HS with CDN1163 (7HSC). The content of phosphorylated CaMKII was normalized to the level of total CaMKII in each sample (**A**). The content of oxidized tropomyosin was normalized to the level of reduced tropomyosin in each sample (**D**). Levels of SERCA1 (**B**), SERCA2 (**C**), CaN (**E**), and calpain-1 (**F**) mRNA were normalized to the RPL19 mRNA expression in each sample. N = 8. * indicates a significant difference from the control, ** indicates a significant difference from the HS group, *p* < 0.05. Original Western blot images are in the Supplementary File S1.

Since SERCA regulates the content of calcium ions in the sarcoplasm, we evaluated the leading makers of calcium-dependent pathways: CaMKII phosphorylation, CaN and calpain-1 mRNA expression. There was a three-fold increase in CaMK IIβ (Thr287) phosphorylation (Figure 2A) and significantly increased mRNA expression of CaN (by 50%) and calpain-1 (by 80%) in the 7HS group when compared with the C group (Figure 2B,C). In the 7HSC group, the CaMK IIβ (Thr287) phosphorylation (Figure 2A) and CaN mRNA expression (Figure 2B) were not statistically different when compared with the C or 7HS group. The upregulation of calpain-1 mRNA expression was significantly attenuated in the 7HSC group when compared with the HS group (Figure 2C). 

mRNA expression of SERCA1 and SERCA2 in the 7HSC group was significantly higher than in the C and 7HS groups (Figure 2D,E). mRNA expression of SERCA1 in the 7HSC group was significantly higher (by 32%) than in the C group (Figure 2D), while SERCA2 mRNA expression was the same in both groups (Figure 2E).

The content of oxidized tropomyosin in the 7HS group was increased by 55% when compared with the C group (Figure 2F). The oxidized tropomyosin content in the 7HSC group was not different from the C group and significantly lower than in the 7HS group (Figure 2F).

### 3.3. The Effects of CDN1163 on Muscle Fiber Size and Fiber Type Composition during Unloading

**Figure 3:**
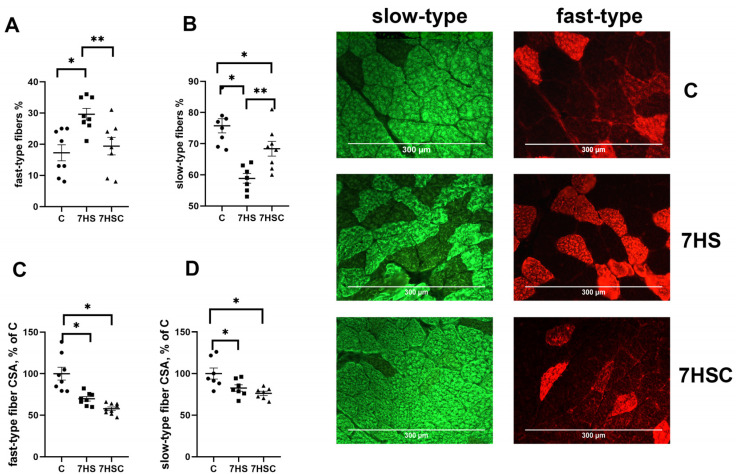
Evaluation of the relative content of fast (**A**) and slow (**B**) muscle fibers and CSA of fast (**C**) and slow (**D**) muscle fibers and a representative picture of fast and slow myosin immunostaining in soleus muscles of control rats (C), rats with 7 days of unloading (7HS), and 7 days of HS with CDN1163 (7HSC). N = 8. * indicates a significant difference from the control, ** indicates a significant difference from the HS group, *p* < 0.05.

Unloading resulted in a decrease in the number of slow fibers in the soleus muscle of 7HS group (by 60%) and an increase in the number of fast fibers (by 22%) when compared with the C group (Figure 3A,B). Treatment with CDN1163 prevented these changes (Figure 3A,B).

The cross-sectional area (CSA) of both fast and slow fibers was significantly lower in both 7HS and 7HSC groups when compared with the C group (Figure 3C,D). Examples of immunostaining with antibodies against slow and fast myosin can be seen in Figure 3.

**Figure 4:**
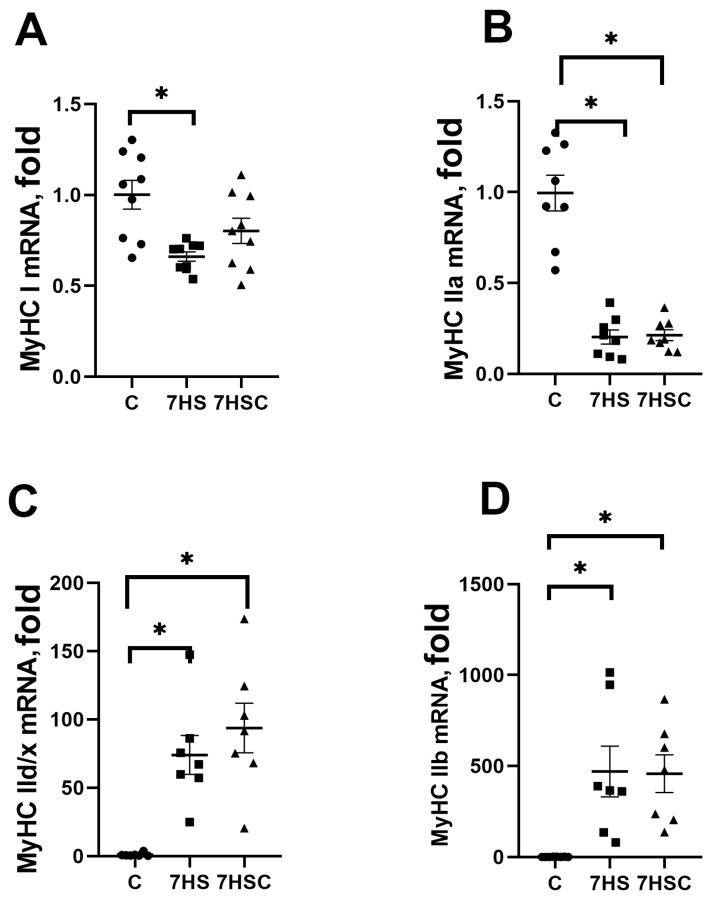
mRNA expression of MyHC Iβ (**A**), MyHC IIa (**B**), MyHC IId/x (**C**), and MyHC IIb (**D**). Myosin in soleus muscles of control rats (C), rats with 7 days of unloading (7HS), and 7 days of HS with CDN1163 (7HSC). Levels of MyHC isoforms mRNA were normalized to the RLP19 mRNA expression in each sample. N = 8. * indicates a significant difference from the control, *p* < 0.05.

The mRNA expression of slow myosin (MyHC Iβ) in the 7HS group was significantly lower (by 44%) than in the C group (Figure 4A). Treatment with CDN1163 diminished the decrease of slow myosin mRNA expression (Figure 4A). The mRNA expression of fast MyHC IIa myosin was decreased in both 7HS (by 81%) and 7HSC (by 78%) groups when compared with the C group (Figure 4B). The mRNA expression of fast MyHC IId/x and MyHC IIb myosin was increased in both 7HS and 7HSC groups when compared with the C group (Figure 4C,D).

### 3.4. The Effects of CDN1163 on NFATc1 Signaling during Unloading

**Figure 5:**
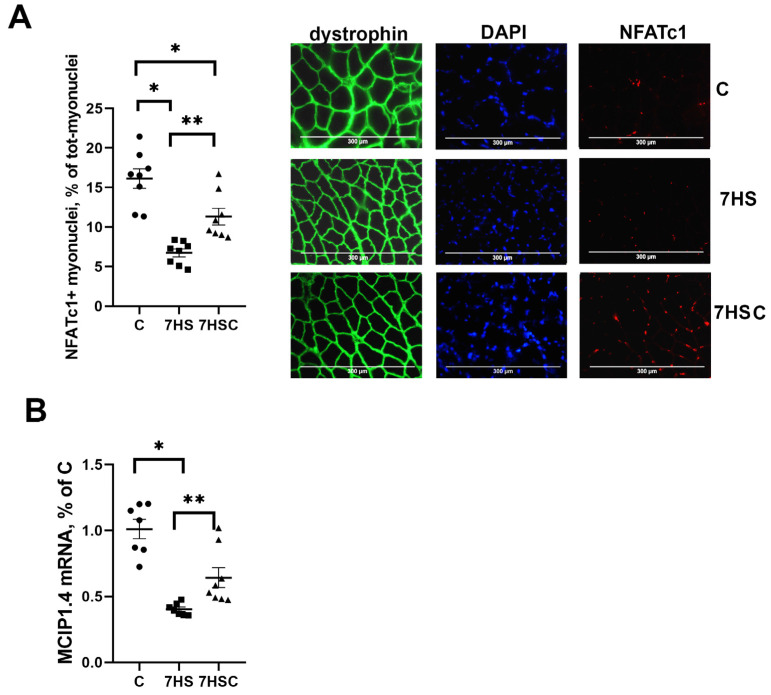
Evaluation of the relative content of NFATc1 using immunostaining (**A**), and the level of MCIP1.4 mRNA expression (**B**) in soleus muscles of control rats (C), rats with 7 days of unloading (7HS), and 7 days of HS with CDN1163 (7HSC). Levels of MCIP1.4 mRNA (**B**) were normalized to RPL19 mRNA expression in each sample. N = 8. * indicates a significant difference from the control, ** indicates a significant difference from the HS group, *p* < 0.05.

NFATc1 is an activator of slow myosin expression in skeletal muscle [40]. The nuclear immunostaining for NFATc1 was decreased by about three-fold by soleus muscle unloading (HS group) when compared with the control muscle (Figure 5A). Treatment with CDN1163 diminished the unloading-induced decrease of the nuclear immunostaining (7HSC group; Figure 5A). The percentage of NFATc1-positive nuclei in the 7HSC group was 38% lower than in the control group (Figure 5A).

MCIP1.4 is a marker of the NFATc1 transcriptional activity [41]. The MCIP1.4 mRNA expression was decreased by about 70% in the HS group when compared with the C group (Figure 5B). The level of MCIP1.4 mRNA expression was significantly higher in the 7HSC group when compared with the HS group (Figure 5B).

**Figure 6:**
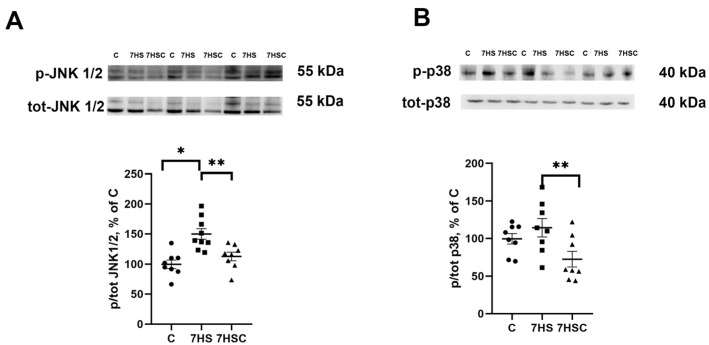
Evaluation of the relative level of JNK1/2 (**A**) and p38 (**B**) phosphorylation in soleus muscles of control rats (C), rats with 7 days of unloading (7HS), and 7 days of HS with CDN1163 (7HSC). The content of p-JNK1/2 (Thr 183, Tyr 185) and p-p38 were normalized to the levels of total JNK ½ and total p38 in each sample, respectively. N = 8. * indicates a significant difference from the control, ** indicates a significant difference from the HS group, *p* < 0.05. Original Western blot images are in the Supplementary File S1.

JNK1/2 and p38 kinases can phosphorylate the NFATc1 transcription factor, causing its removal from the myonuclei and blocking its transcriptional activity [42,43]. Therefore, we tested the levels of JNK1/2 and p38 phosphorylation. The phosphorylation of JNK1/2 kinase was significantly higher in the HS group when compared with the control group (Figure 6A). Treatment with CDN1163 prevented the unloading-induced increase of the JNK1/2 phosphorylation (7HSC group; Figure 6A). The level of p38 phosphorylation was not affected by unloading (HS group; Figure 6B). At the same time, in the 7HSC group, the level of p38 phosphorylation was significantly lower than HS and C groups (Figure 6B).

### 3.5. The Effects of CDN1163 on mRNA Expression of Genes Regulating Mitochondrial Biogenesis during Unloading

**Figure 7:**
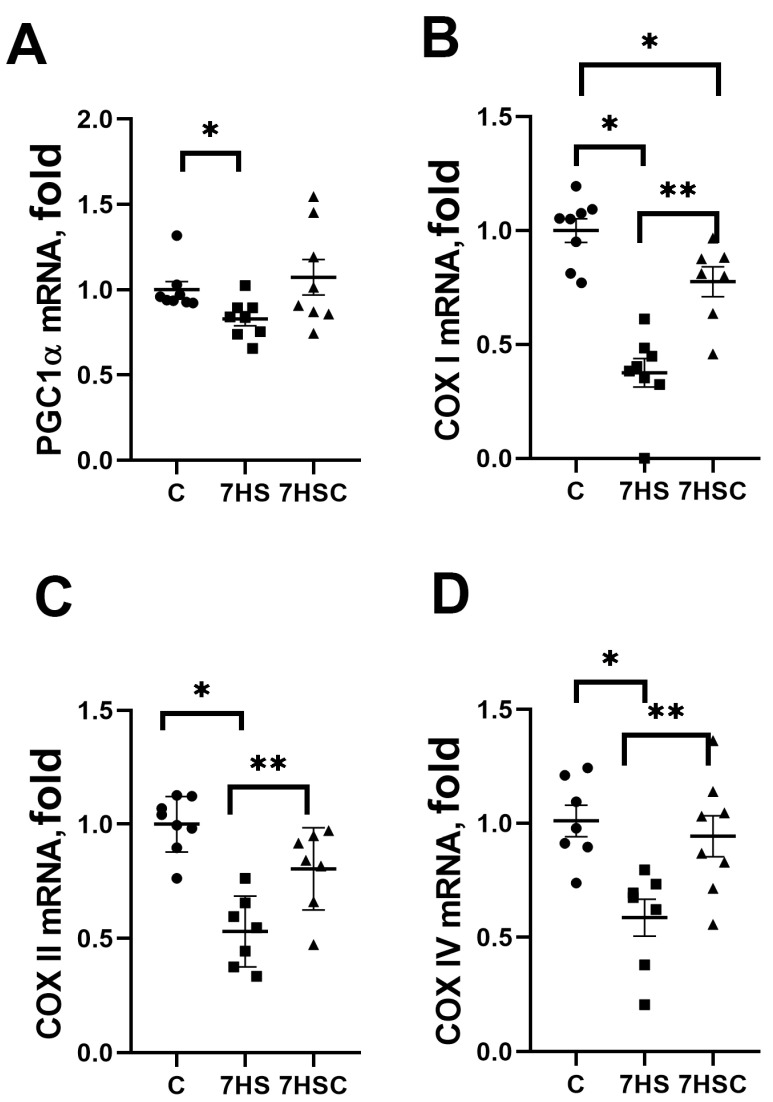
Evaluation of mRNA expression of PGC1a (**A**), COXI (**B**), COXII (**C**), and COXIV (**D**) in soleus muscles of control rats (C), rats with 7 days of unloading (7HS), and 7 days of HS with CDN1163 (7HSC). mRNA expression levels of PGC1α and COX isoforms were normalized to the RPL13 mRNA expression in each sample. N = 8. * indicates a significant difference from the control, ** indicates a significant difference from the HS group, *p* < 0.05.

PGC1α is a critical regulator of mitochondrial biogenesis in skeletal muscle [44]. PGC1α mRNA expression was decreased by 22% in the HS group when compared with the control group (Figure 7A). Treatment with CDN1163 blocked this decrease (7HSC group; Figure 7A).

mRNA expression of COX I was decreased by more than 50% in the HS group when compared with the control group (Figure 7B). In the 7HSC group, the COX I mRNA expression was significantly higher than in the HS group, but it was still lower than in the control group (Figure 7B).

mRNA expression of COX II and COX IV was decreased in the HS group when compared with the control group (Figure 7C and Figure 7D, respectively). Treatment with CDN1163 prevented this decrease (7HSC group; Figure 7C and Figure 7D, respectively).

**Figure 8:**
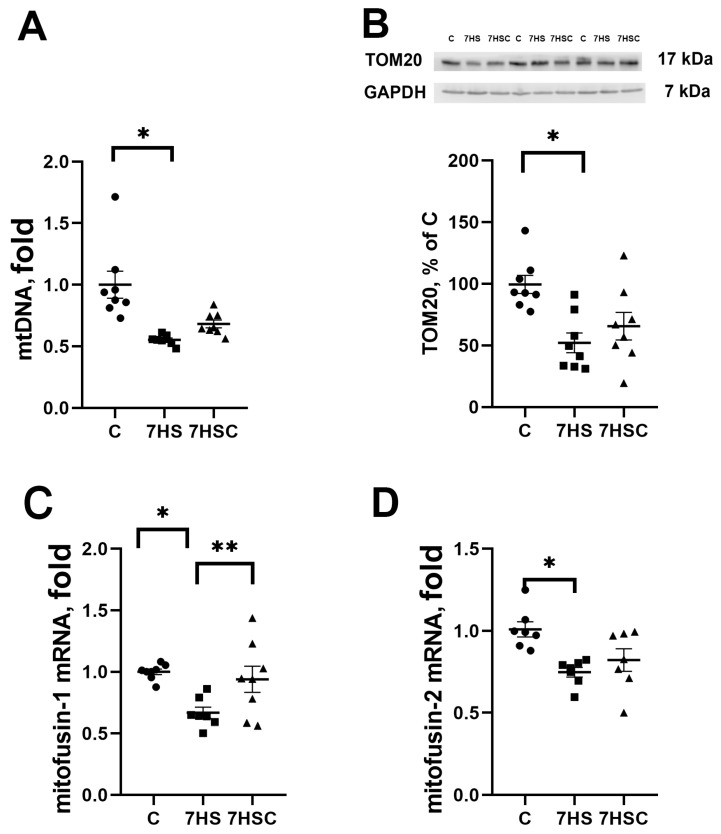
Evaluation of mtDNA content (**A**), TOM20 protein content (**B**), and mitofusin-1 (**C**) and mitofusin-2 (**D**) mRNA expression in soleus muscles of control rats (C), rats with 7 days of unloading (7HS), and 7 days of HS with CDN1163 (7HSC). TOM20 protein expression was normalized to the GAPDH expression in each sample. mRNA expression levels of mitofusins were normalized to the RPL19 mRNA expression in each sample. N = 8. * indicates a significant difference from the control, ** indicates a significant difference from the HS group, *p* < 0.05. Original Western blot images are in the Supplementary File S1.

The mitochondrial DNA content was decreased in both HS and 7HSC groups, although in the 7HSC group, it did not reach statistical significance (Figure 8A). The protein content of structural mitochondrial protein TOM20 was decreased in the HS group when compared with the control group (Figure 8B). This decrease was diminished in the 7HSC group (Figure 8B). The mRNA expression of mitochondrial proteins mitofusin-1 and mitofusin-2 was decreased after muscle unloading (HS group, Figure 8C and Figure 8D, respectively). Treatment with CDN1163 prevented the decrease of mitofusin-1 and diminished the decrease of mitofusin-2 (7HSC group; Figure 8C and Figure 8D, respectively).

### 3.6. The Effects of CDN1163 on the Regulators of Protein Synthesis and Degradation during Unloading

**Figure 9:**
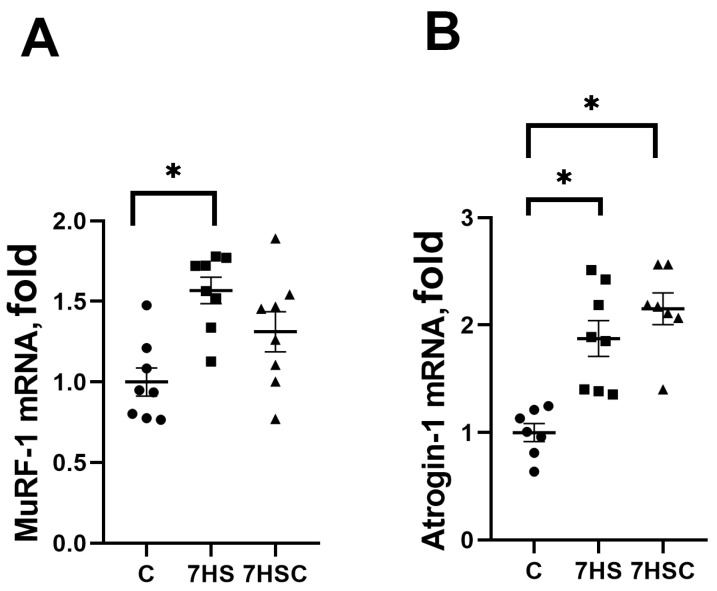
Evaluation of MuRF-1 (**A**) and MAFbx (**B**) mRNA expression in soleus muscles of control rats (C), rats with 7 days of unloading (7HS), and 7 days of HS with CDN1163 (7HSC). mRNA expression levels of MuRF-1 and MAFbx were normalized to the RPL19 mRNA expression in each sample. N = 8. * indicates a significant difference from the control, *p* < 0.05.

In skeletal muscle, protein degradation during unloading is regulated by MuRF-1 and Atrogin-1 (MAFbx) ubiquitin ligases [45]. The mRNA expression of both MuRF-1 and MAFbx was increased in the HS group when compared with the control group (Figure 9A and Figure 9B, respectively). Treatment with CDN1163 slightly diminished the unloading-induced MuRF-1 mRNA expression (7HSC group; Figure 9A), although this decrease did not reach the statistical significance level when compared with the HS group (Figure 9A). MAFbx mRNA expression was not affected by the CDN1163 treatment (7HSC group; Figure 9B).

**Figure 10:**
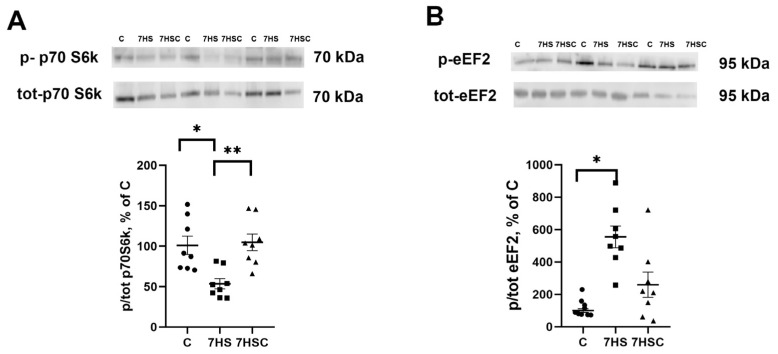
Evaluation of p70S6k (**A**) and eEF2 (**B**) phosphorylation levels in soleus muscles of control rats (C), rats with 7 days of unloading (7HS), and 7 days of HS with CDN1163 (7HSC). The phosphorylation levels of p70S6k and eEF2 were normalized to the levels of non-phosphorylated p70S6k and eEF2 proteins, respectively, in each sample. N = 8. * indicates a significant difference from the control, ** indicates a significant difference from the HS group, *p* < 0.05. Original Western blot images are in the Supplementary File S1.

Protein synthesis is regulated by p70S6k and eEF2 phosphorylation [46]. The level of p70S6k phosphorylation was decreased after unloading two-fold when compared with the control group (Figure 10A). In the 7HSC group, the level of p70S6k phosphorylation was significantly higher than in the HS group and was not different from the control group (Figure 10A). 

The level of eEF2 phosphorylation in the HS group was increased when compared with the control group (Figure 10B). Treatment with CDN1163 slightly diminished the unloading-induced eEF2 phosphorylation (7HSC group; Figure 10B), although it did not reach the statistical significance level when compared with the HS group (Figure 10B).

## 4. Discussion

Unloading-induced skeletal muscle atrophy is regulated by a number of physiological processes. The current study showed that soleus muscle atrophy during seven days of unloading was not affected by the treatment of rats with SERCA activator CDN1163. Soleus muscle weight and CSA of both slow and fast muscle fibers were decreased after seven days of unloading in groups with and without CDN1163 treatment. Unloading-induced muscle atrophy occurs in both male and female animals [47]. The decrease in CSA in the current study was similar to the previously reported data for seven days of unloading [48,49]. The fatigue index was significantly increased after seven days of unloading. Similar data were previously reported for unloaded human and animal muscles [50,51]. At the same time, the fatigue index was significantly improved in the unloaded soleus muscle of rats treated with CDN1163. Improved muscle function after treatment with CDN1163 was also reported for Sod1^−/−^ mice and old mice with sarcopenia [27,28]. The decreased muscle fatigability after treatment with nitrate was also associated with the increase in the submaximal SERCA activity. The authors observed that these properties appear in soleus (expressing type I fibers), but not in EDL (expressing type II fibers) [30]. The maximum force of tetanic contractions was significantly decreased in both HS and HSC groups when compared with the control (data not shown). The specific maximum force was not statistically different between all three groups evaluated in the current study (data not shown).

There are several explanations for the reasons preventing soleus fatigue reduction in the current study after treatment with CDN1163. First, it could be the regulation of sarcoplasmic calcium ions concentrations and consequent regulation of gene expression and oxidative stress. Another contributor could be the regulation of fast/slow myosin expression in unloaded muscle fibers. The third is the regulation of mitochondria in unloaded muscle.

### 4.1. Regulation of Sarcoplasmic Calcium Concentrations and Oxidative Stress during Unloading

Similar to the current study, the increased phosphorylation of CaMK IIβ in skeletal muscle after unloading was reported previously [39]. Intracellular calcium is one of the regulators of CaMKII activity [52,53]. During unloading, the concentration of intracellular calcium is increased [7,37,54]. CaMKII regulates the phosphorylation of several proteins, including AMPK [53,55,56] and some transcription factors [57]. Increased calcium concentrations induce autophosphorylation of CaMK IIβ at Thr 287 [58]. Treatment of rats with CDN1163 in the current study prevented an increase in the level of CaMK IIβ phosphorylation. It can be suggested that this observation is associated with the prevention of an increase in the level of sarcoplasmic calcium in unloaded muscles of rats treated with CDN1163. 

Another calcium-regulated molecule with increased mRNA expression after unloading reported in the current study is CaN. It is a calcium and calmodulin-dependent phosphatase. It was previously reported that CaN content in unloaded muscle is increased simultaneously with the increase in sarcoplasmic calcium concentration [59]. The work of CaN and CaMKII is interrelated [60]. CaN can dephosphorylate specific proteins, initiating their translocation into the nucleus. These dephosphorylated proteins bind to the DNA in the nuclei and regulate the transcription of various genes. In the unloaded muscle of rats treated with CDN1163, CaN mRNA expression was significantly reduced relative to the unloaded muscle of nontreated rats. This suggests that regulated by CDN1163 CaN expression can affect Ca-dependent signaling pathways during unloading.

SERCA regulates the translocation of calcium ions from the sarcoplasm into the sarcoplasmic reticulum. Treatment with CDN1163 significantly increased the mRNA expression levels of SERCA1 and SERCA2 in unloaded muscle. The increased expression of SERCA could contribute to the increased transport of calcium ions from the sarcoplasm into the sarcoplasmic reticulum. Treatment with CDN1163 upregulated the expression of both SERCA isoforms, suggesting a specific effect in unloaded muscle.

Several previous studies used oxidized tropomyosin as an indicator of the oxidative stress index of carboxylation/decarboxylation of muscle proteins and a marker of oxidative tissue damage [61,62,63]. In this study, treatment with CDN1163 prevented an increase in oxidized tropomyosin in unloaded muscle. The data on oxidized tropomyosin presented in the current study correlated well with the previously reported CDN1163-induced decrease in oxidative stress in the muscle of Sod1^−/−^ mice [27] and in mast cells [64]. It was reported that changes in the tropomyosin oxidation in unloaded muscle can modify the sensitivity to calcium and the ability of myofilaments to generate maximum force [61]. Excessive calcium concentration in the sarcoplasm has a direct effect on the ability of myofilaments to generate maximum force. It is achieved through the oxidation of ryanodine receptors, calcium leakage from the sarcoplasmic reticulum, and, therefore, the depletion of the sarcoplasmic reticulum calcium depots [25,65]. The blocked decrease of the unloaded soleus fatigue index after treatment with CDN1163 could be mediated by inhibiting the excessive accumulation of calcium ions and reactive oxygen species in the sarcoplasm.

Phosphorylation of p38 and JNK1/2 kinases is regulated by CaMK II kinase as well as by oxidative stress [66,67]. In the current study, treatment with CDN1163 resulted in a decreased phosphorylation of CaMK II and decreased tropomyosin oxidation in unloaded muscle. This can suggest a decrease in the level of oxidative stress [61,62,63,68]. Therefore, the decrease in phosphorylation of p38 and JNK1/2 kinases in the unloaded muscle of rats treated with CDN1163 reported here can be mediated by a decrease in the level of CaMK II phosphorylation and by decreased oxidative stress.

Treatment with CDN1163 diminished the calpain-1 mRNA upregulation in unloaded muscle in the current study. Calpain-1 is a calcium-dependent protease; therefore, during unloading it can be regulated by increased calcium concentrations.

To summarize, in the current study, treatment with CDN1163 during skeletal muscle unloading downregulated CaMKII phosphorylation, the content of oxidized tropomyosin, mRNA expression of CaN, SERCA1, SERCA2a, and calpain-1.

### 4.2. Regulation of Myosin Composition during Unloading

Treatment with CDN1163 diminished the unloading-related changes in myosin composition. This included both the expression of the slow myosin isoform MyHCIβ and the proportion of slow muscle fibers that are resistant to fatigue. The unloading-induced changes in myosin composition presented here are consistent with the previously reported data [69]. Several mechanisms can be involved in the regulation of myosin composition in the unloaded muscle. The transcription factor NFATc1 can activate the expression of the slow myosin isoform [70,71]. Phosphorylated NFATc1 leaves muscle nuclei and cannot activate the expression of the slow myosin isoform [72]. The current study showed the decreased NFATC1 content in the myonuclei and decreased mRNA expression of MCIP1.4, which is a marker of NFATC1 activity in the unloaded muscle. This is consistent with the previously reported data [73,74]. Treatment with CDN1163 resulted in a higher number of NFATc1-positive myonuclei and a higher level of MCIP1.4 mRNA expression in unloaded muscle when compared with the unloaded muscle without treatment. This can diminish the observed during soleus muscle unloading slow to fast shift in the myosin phenotype.

The phosphorylation of NFATc1 by JNK1/2 and p38 MAP kinases can result in the decrease of its content in the myonuclei and, therefore, a decrease in its transcriptional activity [42,43,75,76]. During unloading, the phosphorylation of JNK1/2 at Thr183/Tyr185 and p38 at Thr180/Tyr182 is increased [77] and this leads to an increase in their activity [78,79]. Pharmacological blocking of p38 activity during unloading prevented a decrease in the MCIP1.4 expression and the content of slow myosin isoform, as well as the decrease in the NFATc1-positive myonuclei [80]. Demonstrated in the current study, there was an increase in JNK1/2 phosphorylation in the unloaded muscle correlates with previously reported data [77]. The absence of an unloading-induced increase in the p38 phosphorylation observed in this study may be explained by the variability in the rates of changes caused by unloading in different animal models. Treatment with CDN1163 led to a significant decrease in the levels of p38 and JNK1/2 phosphorylation observed in the unloaded muscle. Thus, CDN1163-induced inhibition of JNK1/2 and p38 phosphorylation/activity results in the increased accumulation of NFATc1-positive myonuclei and diminished changes of the slow myosin expression in the unloaded soleus muscle.

### 4.3. Regulation of Mitochondria during Unloading

In addition to the effects on the slow myosin composition, the treatment with CDN1163 can also increase the resistance of the soleus muscle to fatigue via regulation of the mitochondrial proteins and DNA content. The current study showed a decrease in the content of mitochondrial DNA and TOM20 protein expression, and decreased PGC1α, COX isoforms, and mitofusins-1 and 2 mRNA expression. TOM20 is a marker of mitochondrial content [81,82]. Observed in the current study, unloading-induced mitochondrial changes are consistent with the previously reported data [39,83,84,85]. Pharmacological activation of SERCA in the liver of obese mice [34] and in human myotubes [86] correlated with the activation of fatty acid utilization and with mitochondrial biogenesis. This correlates well with the data presented here, showing the preserved mitochondrial proteins and DNA content in unloaded soleus muscle treated with CDN1163. 

Excessive accumulation of calcium in the sarcoplasm increases muscle fatigue due to the increased generation of reactive oxygen species by mitochondria, the subsequent increase in the oxidation of ryanodine receptors, calcium leakage from the sarcoplasmic reticulum, and the depletion of muscle fiber calcium depots [24]. A similar mechanism can be involved in the increased soleus muscle fatigability observed during unloading. Treatment with CDN1163 diminishes the unloading-induced accumulation of excessive calcium concentrations and increases accumulation of reactive oxygen species, and this can lead to reduced fatigue in the soleus muscle.

Preserved mitochondrial DNA and protein content in unloaded soleus muscle of CDN1163-treated rats in this study can result in a decreased level of reactive oxygen species that cause mitochondrial stress [87].

### 4.4. The Effects of CDN1163 on the Markers of Signaling Pathways Regulating Protein Synthesis and Protein Degradation during Unloading

In addition to the regulation of NFATc1 accumulation in the myonuclei, p38 and JNK1/2 MAP kinases can regulate the increased expression of MuRF-1 [88,89]. The current study demonstrated an increased mRNA expression of both MuRF-1 and MAFbx E3 ligases in unloaded soleus muscle. This is consistent with earlier studies [39,90]. Treatment with CDN1163 led to a diminished upregulation of the MuRF-1 (but not MAFbx) mRNA expression in unloaded soleus muscle, which may be related to the decreased p38 and JNK1/2 activity. It was previously reported that pharmacologic inhibition of p38 phosphorylation blocks the unloading-induced MuRF-1 (but not MAFbx) mRNA expression in soleus muscle [91]. 

Observed in the current study, the decrease in the level of p70S6k phosphorylation and the increase in the level of eEF2 phosphorylation in unloaded soleus muscle are consistent with the previous observations [49,92].

Increased p70S6k phosphorylation leads to the activation of protein translation, while phosphorylation of eEF2 inhibits its activity and, therefore, leads to a decrease in the level of protein translation [93]. Treatment with CDN1163 prevented the decrease in p70S6k phosphorylation and the increase in eEF2 phosphorylation in unloaded muscle. The effect on eEF2 phosphorylation could be associated with a CDN1163-induced decrease in sarcoplasmic calcium levels since eEF2 phosphorylation is a calcium-dependent process [94].

Despite the observed effects of CDN1163 on protein synthesis and degradation markers in unloaded muscle, it had no effect on soleus muscle atrophy. The observed effect in this study of CDN1163 on mTORC1-dependent p70S6k phosphorylation suggests possible activation of mTORC1 in the unloaded muscle of treated rats. Activation of mTORC1 may lead to improved mitochondrial functions via enhanced mRNA translation encoded by the nuclear genome mitochondrial genes [95]. It is likely that SERCA activation by CDN1163 treatment during unloading may affect mTORC1 and contribute to the improvement of muscle mitochondrial functions.

This research, however, is subject to several limitations. The duration of the experiments was 7 days, and the myofiber atrophy ratio depends on the time of the muscle unloading, so the results may differ after longer or shorter unloading periods [96]. The results of the current study were obtained on soleus muscle, which is a slow-type muscle. The time-course of the unloading-induced fast-type muscles atrophy differs from the slow-type muscles, and the calcium alterations in the fast-type muscles may also differ due to the fast-type-specific mechanisms of calcium regulation [97].

## 5. Conclusions

Treatment of rats for seven days with SERCA activator CDN1163 led to the following changes in the unloaded soleus muscle: (1) it downregulated the level of calcium-dependent phosphorylation of CaMK II and tropomyosin oxidation; (2) it prevented changes in muscle fatigue index by maintaining muscle fiber composition, as well as mitochondrial DNA and protein content; and (3) it preserved some markers of protein synthesis but did not inhibit activation of the proteolysis markers and muscle atrophy.

## Figures and Tables

**Table 1:** PCR primers.

Gene Description	Primer Sequence
Myh7 (MyHC I(β))	5′-ACAGAGGAAGACAGGAAGAACCTAC-3′ 5′-GGGCTTCACAGGCATCCTTAG-3′
Myh2 (MyHC IIa)	5′-TATCCTCAGGCTTCAAGATTTG-3′ 5′-TAAATAGAATCACATGGGGACA-3′
Myh4 (MyHC IIb)	5′-CTGAGGAACAATCCAACGTC-3′ 5′-TTGTGTGATTTCTTCTGTCACCT-3′
Myh1 (MyHC IId/x)	5′-CGCGAGGTTCACACCAAA-3′ 5′-TCCCAAAGTCGTAAGTACAAAATGG-3′
SERCA1	5′-GACTGAGTTTGGGGAACAGCT-3′ 5′-GAGGTGGTGATGACAGCAGG-3′
SERCA2	5′-GAAGCAGTTCATCCGCTACCTCA-3′ 5′-GCAGACCATCCGTCACCAGA-3′
PGC1α	5′-GTGCAGCCAAGACTCTGTATGG-3′ 5′-GTCCAGGTCATTCACATCAAGTTC-3′
Rcan1 (MCIP1.4)	5′-CCGTTGGCTGGAAACAAG-3′ 5′-GGTCACTCTCACACACGTGG-3′
RPL19	5′-GTACCCTTCCTCTTCCCTATGC-3′ 5′-CAATGCCAACTCTCGTCAACAG-3′
Atrogin-1	5′-CTACGATGTTGCAGCCAA-GA-3′ 5′-GGCAGTCGAGAAGTCCAGTC-3′
MuRF-1	5′-GCCAATTTGGTGCTTTTTGT-3′ 5′-AAATTCAGTCCTCTCCCCGT-3′
mtDNA	5′-ATTGGAGGCTTCGGGAACTG-3′ 5′-AGATAGAAGACACCCCGGCT-3′
Mitofusin-1	5′-CCACAGAGCTGGACATCTGG-3′ 5′-GAGAGCCGCTCATTCACCTT-3′
Mitofusin-2	5′-AGTCGGTTGGAAGTCACTGT-3′ 5′-TGTACTCGGGCTGAAAGGAG-3′
COX I	5′-ATTGGAGGCTTCGGGAACTG-3′ 5′-AGATAGAAGACACCCCGGCT-3′
COX II	5′-ATTGGAGGCTTCGGGAACTG-3′ 5′-AGATAGAAGACACCCCGGCT-3′
COXIV	5′-TGGGAGTGTTGTGAAGAGTGA-3′ 5′-GCAGTGAAGCCGATGAAGAAC-3′
CaN A	5′-GCA-CAC-ATA-GAT-GGT-CGG-C-3′ 5′-CAG-GTG-CAT-GCT-TTG-ATC-GC-3′

## Data Availability

Data are available upon reasonable request.

## Supplementary Materials

The following supporting information can be downloaded at: https://www.mdpi.com/article/10.3390/biom13091354/s1, File S1: Original Western blot images.
