# Supplementary material for: The Effect of SERCA Activation on Functional Characteristics and Signaling of Rat Soleus Muscle upon 7 Days of Unloading
Source: Biomolecules. 2023 Sep 6;13(9):1354. doi: 10.3390/biom13091354 (PMC10526198; doi:10.3390/biom13091354)

Oxidized and reduced tropomyosin

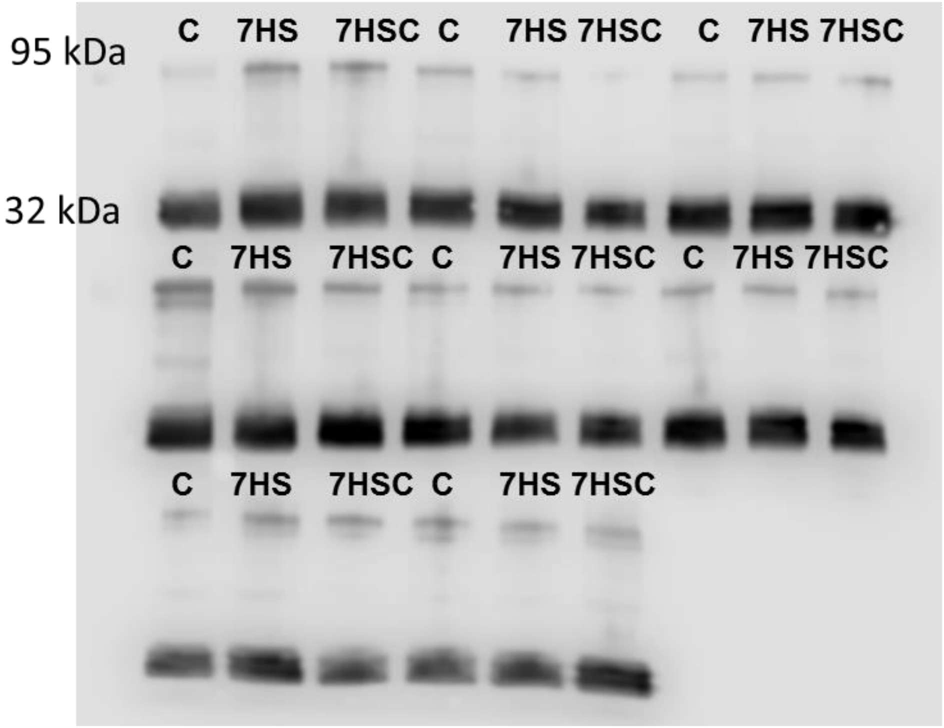

p-CaMK II (beta)

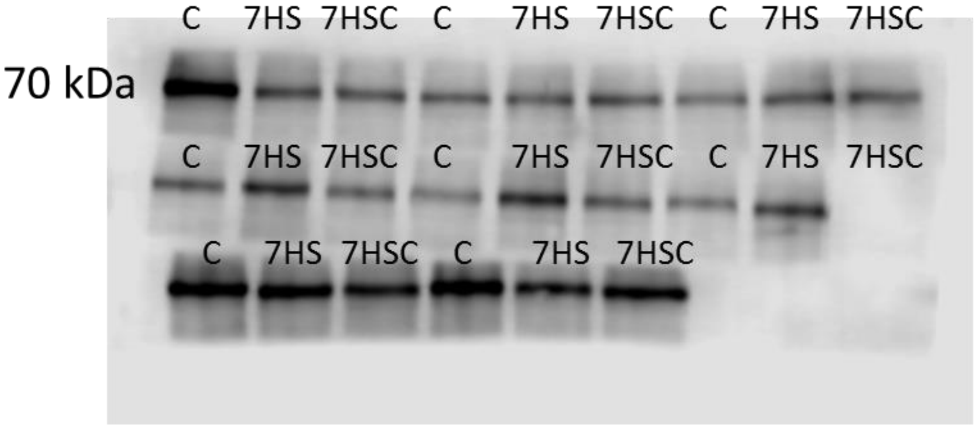

total-CaMK II (beta)

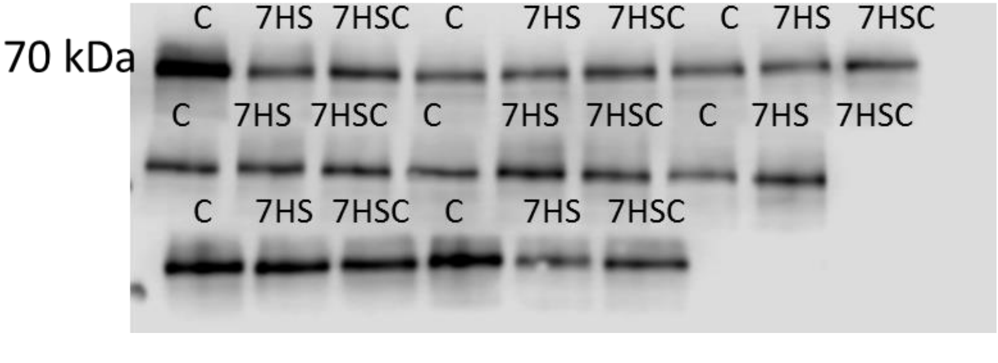

p-JNK1/2

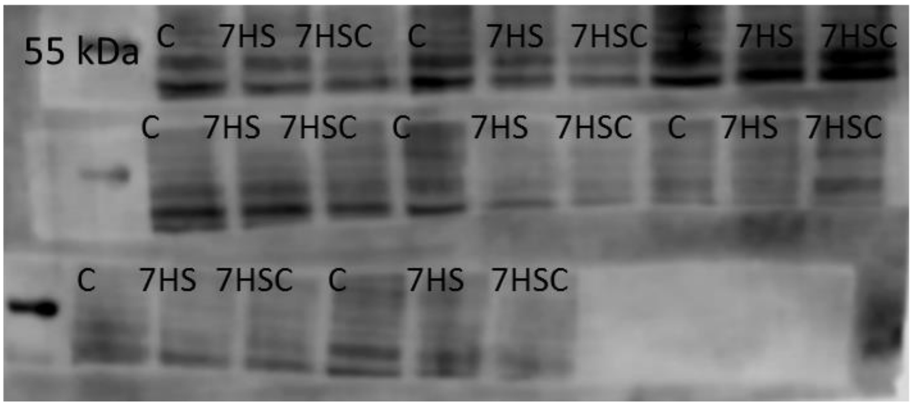

total-JNK1/2

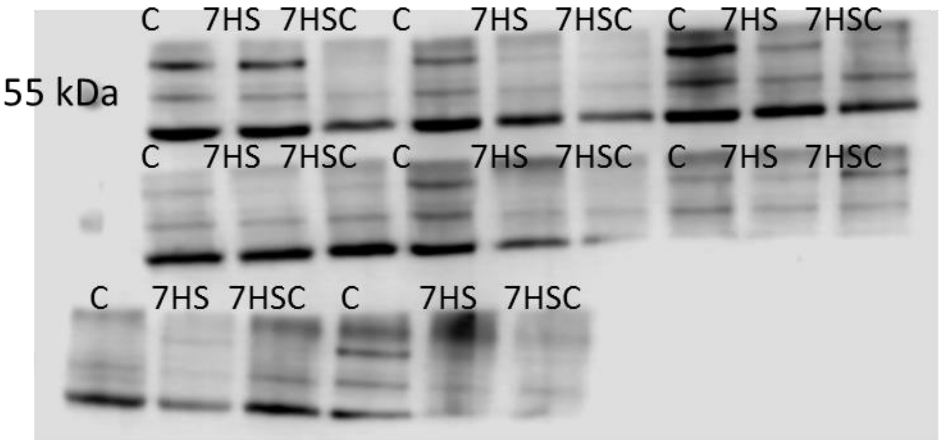

p-p38

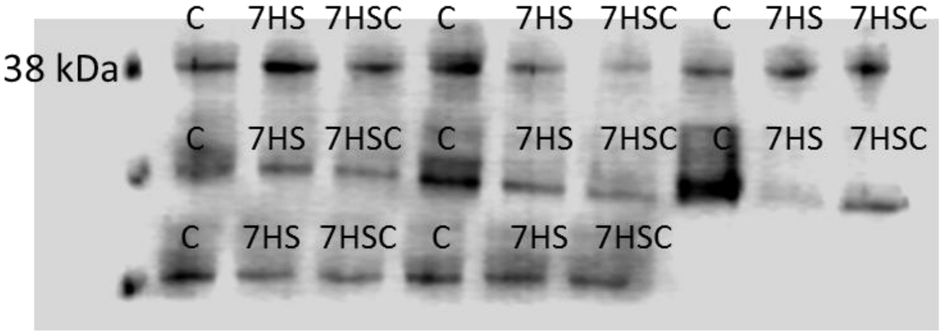

total-p38

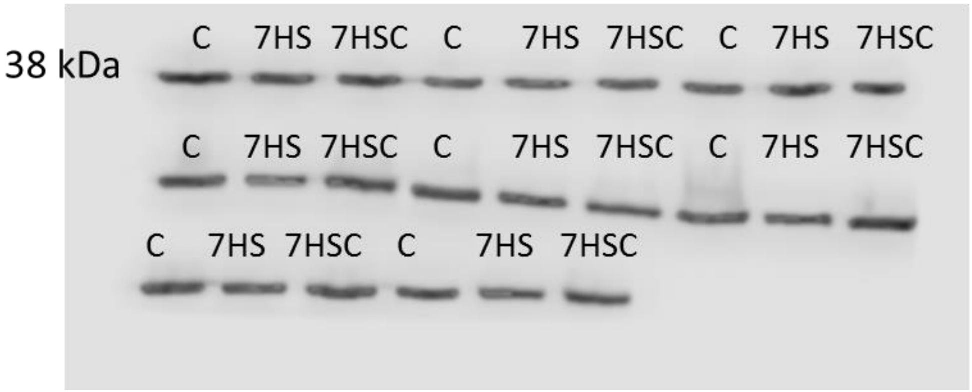

TOM20

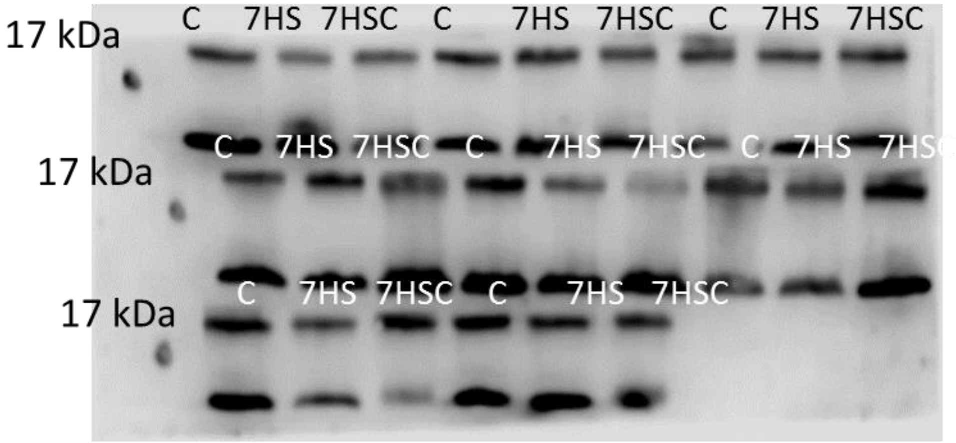

GAPDH

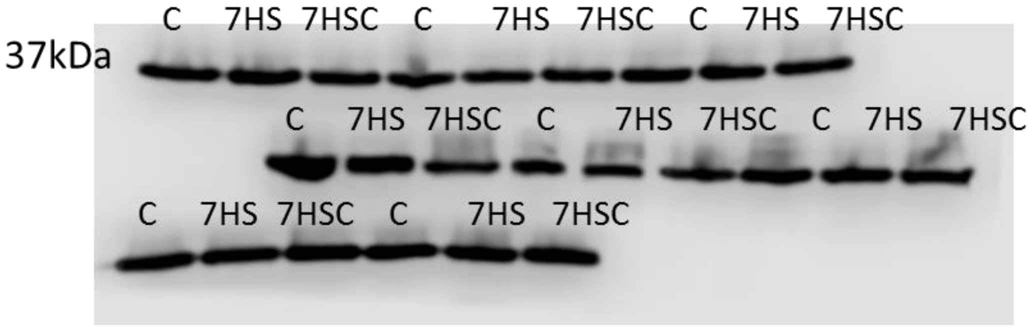

p-p70

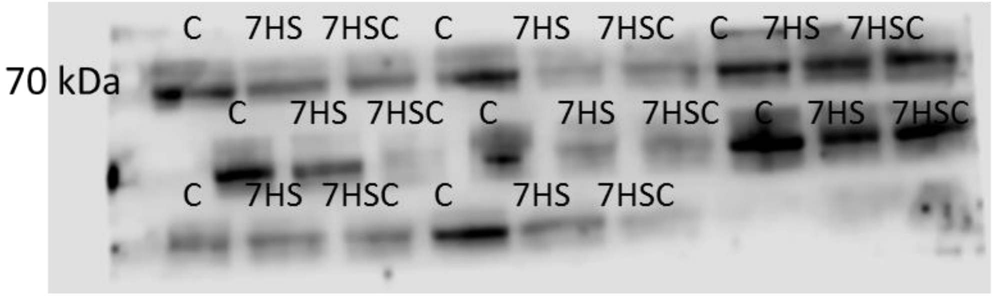

total-p70

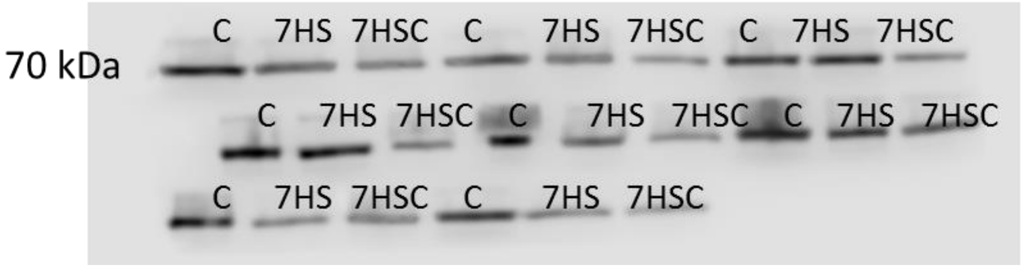

p-eEF2

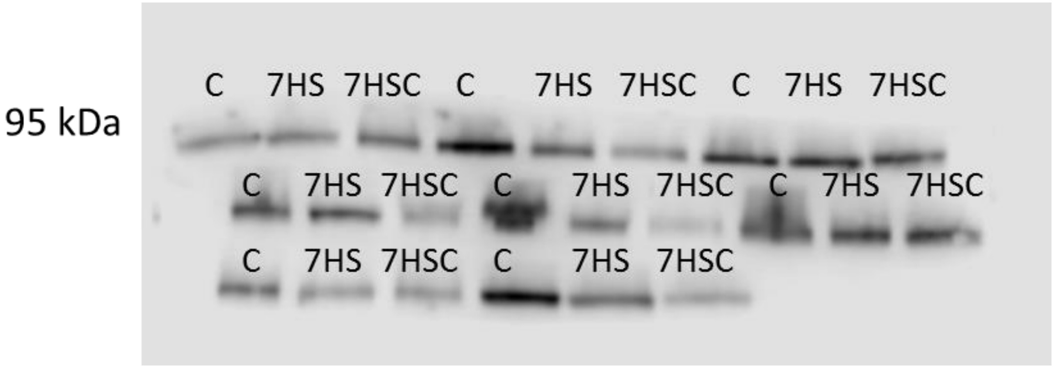

total-eEF2

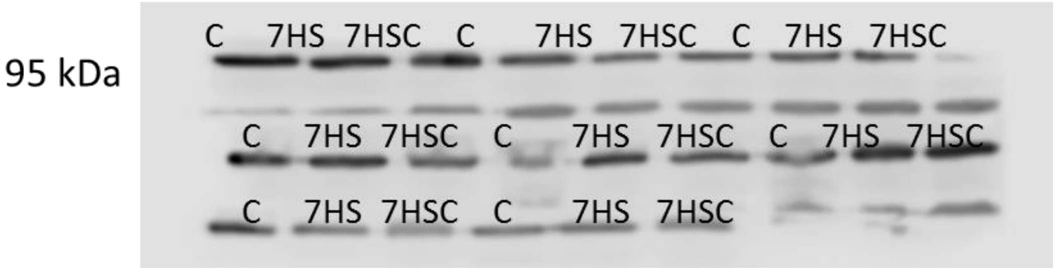

Supplement: Supplementary file 1 [file biomolecules-13-01354-s001.zip › western blot - original images.pdf]
